# An integrated strategy of biological and physical constraints in biological optimization for cervical carcinoma

**DOI:** 10.1186/s13014-017-0784-1

**Published:** 2017-04-04

**Authors:** Ziwei Feng, Cheng Tao, Jian Zhu, Jinhu Chen, Gang Yu, Shaohua Qin, Yong Yin, Dengwang Li

**Affiliations:** 1 Shandong Province Key Laboratory of Medical Physics and Image Processing Technology, Institute of Biomedical Sciences, School of Physics and Electronics, Shandong Normal University, No.88, Wenhua East Road, Lixia District, Jinan, 250014 China; 2 Department of Radiation Oncology, Shandong Cancer Hospital and Institute, No.440, Jiyan Road, Jinan, 250117 China

**Keywords:** Cervical carcinoma, Intensity-modulated radiation therapy, Biology optimization, Physical optimization, Tumor control probability, Normal tissue complication probability

## Abstract

**Background:**

For cervical carcinoma cases, this study aimed to evaluate the quality of intensity-modulated radiation therapy (IMRT) plans optimized by biological constraints. Furthermore, a new integrated strategy in biological planning module was proposed and verified.

**Methods:**

Twenty patients of advanced stage cervical carcinoma were enrolled in this study. For each patient, dose volume optimization (DVO), biological model optimization (BMO) and integrated strategy optimization (ISO) plans were created using same treatment parameters. Different biological models were also used for organ at risk (OAR) in BMO plans, which include the LKB and Poisson models. Next, BMO plans were compared with their corresponding DVO plans, in order to evaluate BMO plan quality. ISO plans were also compared with DVO and BMO plans, in order to verify the performance of the integrated strategy.

**Results:**

BMO plans produced slightly inhomogeneity and less coverage of planning target volume (PTV) (V95=96.79, HI = 0.10: *p* < 0.01). However, the tumor control probability (TCP) value, both from DVO and BMO plans, were comparable. For the OARs, BMO plans produced lower normal tissue complication probability (NTCP) of rectum (NTCP = 0.11) and bladder (NTCP = 0.14) than in the corresponding DVO plans (NTCP = 0.19 and 0.18 for rectum and bladder; *p* < 0.01 for rectum and *p* = 0.03 for bladder). V95, D98, CI and HI values that were produced by ISO plans (V95 = 98.31, D98 = 54.18Gy, CI = 0.76, HI = 0.09) were greatly better than BMO plans (V95 = 96.79, D98 = 53.42Gy, CI = 0.71, HI = 0.10) with significant differences. Furthermore, ISO plans produced lower NTCP values of rectum (NTCP = 0.14) and bladder (NTCP = 0.16) than DVO plans (NTCP = 0.19 and 0.18 for rectum and bladder, respectively) with significant differences.

**Conclusions:**

BMO plans produced lower NTCP values of OARs compared to DVO plans for cervical carcinoma cases, and resulted in slightly less target coverage and homogeneity. The integrated strategy, proposed in this study, could improve the coverage, conformity and homogeneity of PTV greater than the BMO plans, as well as reduce the NTCP values of OARs greater than the DVO plans.

## Background

Intensity-modulated radiation therapy (IMRT) is one of the most common and effective radiotherapy treatment techniques for cervical carcinoma [1–3]. In conventional IMRT plan designing, physical constraints (dose volume parameters) are usually implemented for optimization and plan evaluation. Recently, biological constraints, such as generalized equivalent uniform dose (gEUD), tumor control probability (TCP) and normal tissue complication probability (NTCP), have been used in IMRT planning process [4]. Biological parameters, which applied in biological models to predict the biological effect of tumor and normal tissues, have more direct correlation with treatment outcome than physical parameters [5–10]. Research shows that, with similar target coverage, biological optimization could spare more organs at risk (OARs) than physical optimization [11–18]. However, there are also some disadvantages in biological optimization; such as highly inhomogeneous target dose distributions [11, 12, 18]. Thus, additional physical constraints involved in the biological optimization have been proposed in several studies in order to improve the target dose homogeneity and conformity [4, 12, 15–18]. Kan et. al indicated that the amount of hot spots and the maximum doses within targets could be substantially reduced by adding the physical constraints in biological optimization [18].

The biological planning module in Eclipse System (Varian Medical Systems, Palo Alto, CA) could be used for biological optimization with biological constraints like EUD, TCP and NTCP, as well as physical constraints. Kan et. al found that the target coverage and conformity, produced by biological planning module of Eclipse system, were comparable with physical optimization, but spared more parotid glands [18]. Additionally, the research also outlined the limitations of the biological planning module and stated that it would produce more hot spots in PTV for the current module, for it does not allow the users to specify the priority of physical constraints [18]. In the study, additional physical constraints were implemented in the biological planning module to assistant the biological optimization. There are no further studies on how to effectively integrate the biological constraints and physical constraints in the biological planning module for producing better plan quality.

In this study, the primary purpose is to investigate whether the biological optimization could be an alternative method to the conventional physical optimization for cervical carcinoma, by comparing plan qualities between the dose volume based optimization (DVO, only physical constraints were used in the optimization) and biological model based optimization (BMO, biological constraints were used in the optimization with several assistant physical constraints). Furthermore, the IMRT plan quality produced by BMO were tested and evaluated by different biological models. Based on both dosimetrical and biological endpoints consideration, we propose a novel strategy, integrated strategy based optimization (ISO, physical and biological constraints were motivationally integrated in the optimization) to improve plan quality. And all IMRT plans were evaluated by physical and biological indices.

## Methods

### Patient selection

Twenty patients with cervical carcinoma were selected for this study, which pathology diagnosis ranged from stage IIIB to IV. The computed tomography (CT) images (3 mm slice thickness, 512 × 512 pixels/slice) were used for acquiring volumetric anatomical data and analyzing dosimetry comparison. This study was a retrospective study, and patient treatment outcomes were not involved.

### Treatment planning

The Eclipse treatment system (Version 13.5, Varian Medical System Inc, Palo Alto, CA) was used to generate all IMRT plans optimized by biological and physical constraints in this study. Specifically, 6 MV photon beams of a linear accelerator (Trilogy, Varian Medical Systems) with 120 multi-leaf collimator was applied.

The clinical target volume (CTV) of each patient, including the primary tumor area, uterus, and the pelvic and para-aortic lymph nodes, was contoured by an experienced oncologist. The corresponding planning target volume (PTV) was generated by expanding 0.5 cm from CTV symmetrically. The OARs included rectum, bladder, femur-heads and small bowel in this study. In order to improve the target dose conformity, the assistance organ Body-PTV (B-P) was defined as the body volume in the CT data set minus the PTV leaving a 0.3 cm gap. B-P was used in all IMRT optimization in order to standardize the optimized constraints.

The prescribed dose to PTV was set to 54 Gy with 30 fractions. All plans were normalized to a mean dose of PTV (and isodose 95% was set to the prescribed dose) in order to make plan comparisons valid. For all patients, the IMRT plans were created using 7 evenly distributed coplanar fields (every 51 °). The same photon beam settings of DVO, including number and orientations of beams, beam energy and iso-center position, were used for corresponding BMO and ISO. This is to eliminate the differences of plan quality that result from the variation in radiation beam parameters.

The optimization process was applied by two-step algorithm in the Eclipse planning system [19, 20]. All dose calculations were performed with the anisotropic analytical algorithm (AAA) with a calculation grid of 2.5 mm [21]. All plans were generated by a singular, experienced dosimetrist in order to avoid the variation of plan quality.

### Dose volume optimization

In DVO plans, the clinical requirements of dose volume for structures were listed in Table 1. According to our institution planning protocol for physical optimization, 95% of PTV should receive at least the prescribed dose, while reducing the dose received by OARs. In this aspect, the higher priorities were to give enough dose coverage to PTV and reduce the maximum doses to B-P; while the dose volume constraints for OARs were given lower priority. The physical constraints were listed in Table 2.

**Table 1:** Clinical requirements to target and OARs

Structures		Constraints	
		Mean dose	Dose volume
PTV		-	At least 95% volume of PTV to receive the prescribed dose
OAR	Rectum	<35 Gy	Less than 40% volume to exceed 40 Gy
	Bladder	<35 Gy	Less than 40% volume to exceed 40 Gy
	Femur-heads	<25 Gy	Less than 5% volume to exceed 40 Gy

**Table 2:** The physical constraints used in DVO

Structures		Constraints	Parameters
PTV		Min dose	54 Gy
		Dose/volume	V_97_ > 54.5 Gy
		Dose/volume	V_3_ < 57.5 Gy
		Max dose	58 Gy
OAR	Rectum	Dose/volume	V_40_ < 35 Gy, and two extra dose/volume constraints for reducing the mean dose
	Bladder	Dose/volume	V_40_ < 35 Gy, and two extra dose/volume constraints for reducing the mean dose
	Femur-heads	Dose/volume	V_5_ < 35Gy, and two extra dose/volume constraints for reducing the mean dose
B-P^a^		Max dose	50 Gy

^a^The assistance organ Body-PTV (B-P) was defined as the body volume in the CT data set minus the PTV leaving a 0.3 cm gap

Additionally, in the optimization process, the hot or cold spots within PTV and the high dose region of normal tissue should be re-countered and defined as virtual organs after each optimization cycles. Then, dose volume constrains with appropriate priority were set to these virtual organs, and re-optimization was applied in order to achieve the clinical requirements and produce the desired dose distribution.

### Biological model optimization

The BMO plans were generated on biological planning module in Eclipse planning system. In this module, biological models like Poisson-LQ and LKB were involved for biological optimization. The details of these biological models could be found in previous studies [22–29]. Additionally, gEUD, a semi-biological constraint, was implemented in this module.

The parameters of the TCP and NTCP functions in the biological planning module, such as the ${D}_{50}$, n, m, $\gamma$ and $\alpha /\beta$ values, could be adjusted manually, and these adjustments would have a significant impact on plan quality. Thus, in this study, we just applied the default parameters, given in the module, which was collected from previous retrospective clinical research [26–29].

For qualifying the BMO plans qualities and illuminating the influence caused by different biological models, several biological models (which were pre-set in biological planning module of Eclipse) were implemented. In this research, TCP Poisson-LQ model (cervix-IIIB, IV [26]) was applied for PTV. Different biological models were used for OARs: the rectum used the NTCP Poisson-LQ model (Necrosis/Stenosis [27], as a superscript of “cr” ) and LKB model (Late rectal bleeding, grade > =2 [28], as a superscript of “br”; and Late effects, grade > =3 [29], as a superscript of “ar”), the bladder used the NTCP Poisson-LQ model (Contracture [27], as a superscript of “bb”) and the NTCP LKB model (Late effects, grade > =3 [29], as a superscript of “ab”), and the femur-heads used the NTCP Poisson-LQ model (Necrosis [27]). The pre-set specific values of these biological parameters could be found in Table 3. For comparing different rectum biological models, BMO plans were generated by “ar” “br” and “cr” models respectively with fixed bladder model “ab”. And so did bladder. Fixed the “ar” rectum model, the corresponding BMO plans were generated by “ab” and “bb” bladder models respectively. The small bowel was not considered in the optimization, but the NTCP Poisson-LQ model (Obstruction/Perforation [27]) of small bowel, used for biological evaluation, was also listed in Table 3.

**Table 3:** The biological constraints and additional physical constrains used in BMO

Structures		Model or constraints	Parameters
PTV		TCP Poisson-LQ	${D}_{50}$ =77.6Gy, $\gamma$ =1.2, $\alpha /\beta$ =10Gy
		Target EUD	D = 54Gy, a = 0.1
		Max Dose	D = 58Gy
		Uniformity constraint	Std. dev = 2%
OARs	Rectum	NTCP LKB^ar^	${D}_{50}$ =80Gy, $\gamma$ =3.9, n = 0.06, m = 0.15
		NTCP LKB^br^	${D}_{50}$ =81.8Gy, $\gamma$ =3, n = 0.29, m = 0.22
		NTCP Poisson-LQ^cr^	${D}_{50}$ =80Gy, $\gamma$ =2.2, $\alpha /\beta$ =3Gy, s = 1
		Max EUD	D (variable), a = 1
	Bladder	NTCP LKB^ab^	${D}_{50}$ =62Gy, $\gamma$ =6, n = 0.13, m = 0.11
		NTCP Poisson-LQ^bb^	${D}_{50}$ =80Gy, $\gamma$ =3, $\alpha /\beta$ =3Gy, s = 0.18
		Max EUD	D (variable), a = 1
	Femur-heads	NTCP Poisson-LQ	${D}_{50}$ =65Gy, $\gamma$ =2.7, $\alpha /\beta$ =3Gy, s = 1
		Max EUD	D (variable), a = 1
	Small bowel^a^	NTCP Poisson-LQ	${D}_{50}$ =53.6Gy, $\gamma$ =2.3, $\alpha /\beta$ =3Gy, s = 1.5
B-P		Max Dose	D = 50Gy

The superscripts of NTCP models denote the different biological models of rectum and bladder which will be used in the results section

^a^The small bowel was not involved in biological optimization. The biological parameter was just used for the biological evaluation

Due to the similar results of dose distribution from different biological models, only LKB model “ar” for rectum and LKB model “ab” for bladder were implemented in the comparison of DVO, BMO and ISO plans.

Besides the biological constraints, additional physical constraints, such as the Max Dose and Uniformity constraint, were also applied to PTV in BMO plans to reduce hot spots and improve the target dose heterogeneity. A Max Dose constraint was also assigned to the normal tissues represented by B-P in order to improve target conformities. Because the NTCP parameters could not achieve the acceptable doses levels purely, the Max EUD constraints were implemented in optimization process to further reduce the doses to OARs. These additional physical constraints or semi-biological constraints that were used in biological planning module were also listed in Table 3.

### Integrated strategy optimization

The biological planning module in the Eclipse not only allowed users to implement physical constraints, but also permitted users to arbitrarily apply either physical or biological constraints for structures. In previous research [4, 12, 15–18], physical constraints were only considered as an assistant method to compensate the disadvantage of biological constraints in biological optimization process.

However, based on our comparison of DVO and BMO plans above, a new strategy was proposed: physical constraints were only used for PTV, while biological constraints were only applied for OARs in the biological planning module, to improve the quality of plans generated by the biological planning module. Specifically, in ISO planning, the same physical constraints applied in DVO (constraints for PTV in Table 2) were used for PTV, while the same biological constraints like LKB model “ar” for rectum, LKB model “ab” for bladder, and gEUD constraints applied in BMO (constraints for OARs in Table 3) were used for OARs.

### Plan evaluation and statistics analysis

All IMRT plans were evaluated by physical indices and biological indices.

For the dosimetric evaluation, the following indices were reported: V_95_, the target volume received the prescribe dose as displayed on the cumulative DVH; the maximum and minimum doses, represented by the doses received by 2% (D_2_) and 98% (D_98_) of the target volume respectively. Additionally, the homogeneity index (HI) and conformity index (CI) of target were also calculated by the following: $\mathrm{H}\mathrm{I}=\left({D}_2-{D}_{98}\right)/{D}_{mean}$ [30] and $\mathrm{C}\mathrm{I}={\left({V}_{PTV* Pre}\right)}^2/\left({V}_{PTV}*{V}_{Pre}\right)$ [31]. For OARs, the mean dose (D_mean_) and max dose (D_max_) of rectum and bladder were reported, and the volume of these OARs receiving more than 20, 30, 40 and 50 Gy (V_20_, V_30_, V_40_, V_50_) were analyzed [32, 33].

The radiobiological evaluation was calculated using the available DVHs. TCP was evaluated for PTV, and the NTCP was used for normal tissues. Specifically, the TCP and NTCP models used in the calculation were listed in Table 3. LKB model “ar” for rectum and LKB model “ab” for bladder were used for plan evaluation in the comparison of DVO, BMO and ISO. While in the comparison of different biological models, the rectum and bladder models was applied respectively.

For statistical analysis, a paired t-test was used to compare the IMRT plans. A *p* value of *p* < 0.05 is considered statistically significant. All statistical tests were two-tailed and were performed using R-project software.

## Results

### Comparison between DVO plans and BMO plans

The physical indices of PTV and OARs extracted from DVO plans and BMO plans were shown in Table 4. For D_98_ and V_95_, BMO plans produced lower values (D_98_ = 53.42 Gy, V_95_ = 96.79%) with significant differences (*p* < 0.01) than DVO plans (D_98_ = 54.57 Gy, V_95_ = 99.13%). Concerning PTV homogeneity, better results were obtained for DVO plans (HI = 0.08) with significant differences (*p* < 0.01) in comparison to BMO plans (HI = 0.10). And the same result was observed in the conformity index case. DVO plans produced higher CI value (CI = 0.82) compared to BMO plans (CI = 0.71). Furthermore, there were no regular results between DVO plans and BMO plans in the sparing of rectum and bladder, according to the value of V_20_, V_30_, V_40_ and D_mean_. However, D_max_ values of rectum and bladder of DVO plans (D_max_ = 56.55 Gy and 58.95 Gy for rectum and bladder, respectively) were higher than the corresponding BMO plans (D_max_ = 54.29 Gy and 58.22 Gy for rectum and bladder, respectively) with significant differences (*p* < 0.01 for both rectum and bladder). And DVO plans produced higher V_50_ values of rectum (V_50_ = 6.16%) with significant differences (*p* < 0.01) when compared to BMO plans (V_50_ = 4.35%).

**Table 4:** Evaluation of DVO plans, BMO plans and ISO plans

Structures		Parameters	DVO	BMO	ISO	P1	P2	P3
PTV		D_98_ (Gy)	54.57 ± 0.46	53.42 ± 0.36	54.18 ± 0.38	<0.01	<0.01	<0.01
		D_2_ (Gy)	58.82 ± 0.22	58.72 ± 0.28	58.89 ± 0.26	0.27	0.12	0.18
		V_95_ (%)	99.13 ± 0.82	96.79 ± 0.81	98.31 ± 0.91	<0.01	<0.01	<0.01
		HI	0.08 ± 0.01	0.10 ± 0.01	0.09 ± 0.01	<0.01	<0.01	<0.01
		CI	0.82 ± 0.04	0.71 ± 0.05	0.76 ± 0.04	<0.01	<0.01	<0.01
		TCP	15.39 ± 0.06	15.55 ± 0.58	15.39 ± 0.05	0.22	0.23	0.97
OAR	Rectum	V_20_ (%)	97.55 ± 3.41	95.21 ± 6.98	92.66 ± 6.36	0.10	<0.01	<0.01
		V_30_ (%)	63.43 ± 10.06	69.67 ± 12.80	60.29 ± 12.76	0.01	<0.01	0.17
		V_40_ (%)	26.46 ± 10.90	24.35 ± 12.44	24.24 ± 10.14	0.13	0.93	0.07
		V_50_ (%)	6.16 ± 5.63	4.35 ± 5.82	7.62 ± 10.66	<0.01	0.02	0.31
		D_max_ (Gy)	56.55 ± 2.06	54.29 ± 2.93	56.00 ± 2.01	<0.01	<0.01	0.13
		D_mean_ (Gy)	34.62 ± 2.81	34.49 ± 2.68	33.49 ± 2.59	0.66	<0.01	<0.01
		NTCP	0.19 ± 0.17^ar^	0.11 ± 0.12^ar^	0.14 ± 0.13^ar^	<0.01	<0.01	<0.01
	Bladder	V_20_ (%)	95.35 ± 4.50	93.90 ± 7.13	91.29 ± 7.79	0.35	<0.01	0.03
		V_30_ (%)	62.10 ± 8.71	63.32 ± 8.90	58.06 ± 8.12	0.47	<0.01	<0.01
		V_40_ (%)	29.08 ± 7.07	30.27 ± 7.29	29.68 ± 6.71	0.25	0.28	0.54
		V_50_ (%)	9.79 ± 5.96	8.86 ± 6.44	9.83 ± 6.80	0.07	0.02	0.93
		D_max_ (Gy)	58.95 ± 1.38	58.22 ± 1.75	58.63 ± 1.72	<0.01	0.09	0.09
		D_mean_ (Gy)	34.29 ± 2.19	34.65 ± 2.22	33.92 ± 2.13	0.15	<0.01	0.12
		NTCP	0.18 ± 0.20^ab^	0.14 ± 0.19 ^ab^	0.16 ± 0.20^ab^	0.03	0.15	<0.01
	Small bowel	V_20_ (%)	52.02 ± 8.45	51.37 ± 9.77	51.77 ± 8.90	0.44	0.35	0.41
		V_45_ (%)	0.87 ± 1.48	0.83 ± 1.62	0.84 ± 1.79	0.32	0.26	0.30
		NTCP	0.94 ± 1.53	0.89 ± 1.74	0.90 ± 1.88	0.39	0.23	0.28
B-P		D_max_ (Gy)	57.59 ± 3.05	58.39 ± 1.88	58.04 ± 2.41	0.17	0.27	0.36

The *p* value p1, p2, p3 were represented of the *p* value between DVO and BMO, BMO and ISO, DVO and ISO, respectively. The superscripts of NTCP values denote the biological models of rectum and bladder which listed in Table 3 used for IMRT optimization. ar: LKB with ${D}_{50}$ =80Gy, $\gamma$ =3.9, N = 0.06, M = 0.15 for rectum; ab: LKB with ${D}_{50}$ =62Gy, $\gamma$ =6, N = 0.13, M = 0.11 for bladder

Table 4 also summarized the biological evaluation results for PTV and principal OARs. Considering the TCP value, BMO plans indices were generally higher compared to DVO plans without significant differences. However, the rectum and bladder NTCP values of BMO plans (NTCP = 0.19 and 0.18 for the rectum and bladder, respectively) were lower than DVO plans (NTCP = 0.11 and 0.14 for the rectum and bladder, respectively) with *p* < 0.01 and *p* = 0.03 respectively. Additionally, the NTCP values of small bowel were 0 in all plans.

### Different biological models

In Table 5, we reported the average NTCP values of OARs optimized by different biological models respectively. The superscripts of NTCP values in the table denote the different biological models of rectum or bladder which listed in Table 3 used in IMRT optimization. In this comparison, when different biological models used to one OAR, we keep the biological models of other OARs fixed. The details of biological models used could be found in the footnote of Table 5. Actually, the different IMRT plans optimized by different NTCP models for one OAR produced very similar dose distribution. But the NTCP values between these biological models shown different behavior. In rectum case, the NTCP value of model ‘ar’ (NTCP = 0.11) was between the model ‘br’ (NTCP = 0.33) and model ‘cr’ (NTCP = 0.01) with significant differences (‘ar’ vs. ‘br’: *p* < 0.01; ‘ar’ vs. ‘cr’: *p* < 0.01). In bladder case, the NTCP value of model ‘ab’ (NTCP = 0.14) was greater than that of model ‘bb’ (NTCP = 0.00) with *p* = 0.03.

**Table 5:** Evaluation of BMO plans with different biological models

Biological comparison	Structures	NTCP value	NTCP value	Paired t-test p
Rectum: ar VS. br	Rectum	0.11 ± 0.12^ar^	0.33 ± 0.19^br^	<0.01
	Bladder	0.14 ± 0.19^ab^	0.14 ± 0.19^ab^	-
Rectum: ar VS. cr	Rectum	0.11 ± 0.12^ar^	0.01 ± 0.02^cr^	<0.01
	Bladder	0.14 ± 0.19^ab^	0.14 ± 0.19^ab^	-
Bladder: ab VS. bb	Rectum	0.11 ± 0.12^ar^	0.11 ± 0.12^ar^	-
	Bladder	0.14 ± 0.19^ab^	0.00 ± 0.00^bb^	<0.01

The superscripts of NTCP values denote the different biological models of rectum and bladder which listed in Table 3 used for IMRT optimization. ar: LKB with ${D}_{50}$ =80Gy, $\gamma$ =3.9, N = 0.06, M = 0.15 for rectum; br: LKB with ${D}_{50}$ =81.8Gy, $\gamma$ =3, N = 0.29, M = 0.22 for rectum; cr: Poisson-LQ with ${D}_{50}$ =80Gy, $\gamma$ =2.2, $\alpha /\beta$ =3Gy, s = 1 for rectum; ab: LKB with ${D}_{50}$ =62Gy, $\gamma$ =6, N = 0.13, M = 0.11 for bladder; bb: Poisson-LQ with ${D}_{50}$ =80Gy, $\gamma$ =3, $\alpha /\beta$ =3Gy, s = 0.18 for bladder

### The evaluation of ISO plans

As shown in the Table 4, V_95_, D_98_ and CI value produced by ISO plans (V_95_ = 98.31%, D_98_ = 54.18 Gy, CI = 0.76) were lower than DVO plans (V_95_ = 99.13%, D_98_ = 54.57 Gy, CI = 0.82) with significant differences (*p* < 0.01 for all indices). ISO plans indices were higher than BMO plans’ (V_95_ = 96.79%, D_98_ = 53.42 Gy, CI = 0.71) with significant differences (*p* < 0.01 for all indices). And these differences were much bigger than the differences between ISO plans and DVO plans. The target homogeneity of ISO plans (HI = 0.09) slightly improved when compared with BMO plans (HI = 0.10), but is slightly worse than DVO plans (HI = 0.08). No significant differences were found in TCP values among these plans.

According to Table 4, ISO plans generate the lowest V_20_ and D_mean_ values (V_20_ = 92.66%, D_mean_ = 33.49 Gy) of rectum, compared with the other two types plan (V_20_ = 97.55% and D_mean_ = 34.62 Gy for DVO plans, respectively; V_20_ = 95.21% and D_mean_ = 34.49 Gy for BMO plans, respectively). The same behavior was observed in V_30_ of bladder.

Additionally, the D_max_ value of rectum and bladder generated by ISO plans (D_max_ = 56.00 Gy and 58.63 Gy for rectum and bladder, respectively) were generally lower than those generated by DVO plans (D_max_ = 56.55 Gy and 58.95 Gy for rectum and bladder, respectively), and higher than those of BMO plans (D_max_ = 54.29 Gy and 58.22 Gy for rectum and bladder, respectively) with significant differences (*p* < 0.01 for all cases). In other words, ISO plans generated an intermediate D_max_ value of rectum and bladder between DVO plans and BVO plans. The same situation occurred in the NTCP. Specifically, the NTCP value of rectum produced by ISO plans (NTCP = 0.14) was an intermediate value between DVO plans (NTCP = 0.19) and BMO plans (NTCP = 0.11) with significant differences. Furthermore, the ISO plans produced lower NTCP value (NTCP = 0.16) of bladder compared to the DVO plans (NTCP = 0.18). No significant difference was observed between ISO plans and BMO plans.

The results of small bowel which was not considered in optimization, was also listed in Table 4. There were no significant differences of V_20_, V_45_ and NTCP values of small bowel among these three plans. Otherwise, the D_max_ value of B-P generated by ISO plans (D_max_ = 58.04 Gy) were a median value without significant differences, compared with the one generated by DVO plans (D_max_ = 57.59 Gy) and BMO plans (D_max_ = 58.39 Gy).

**Fig. 1:**
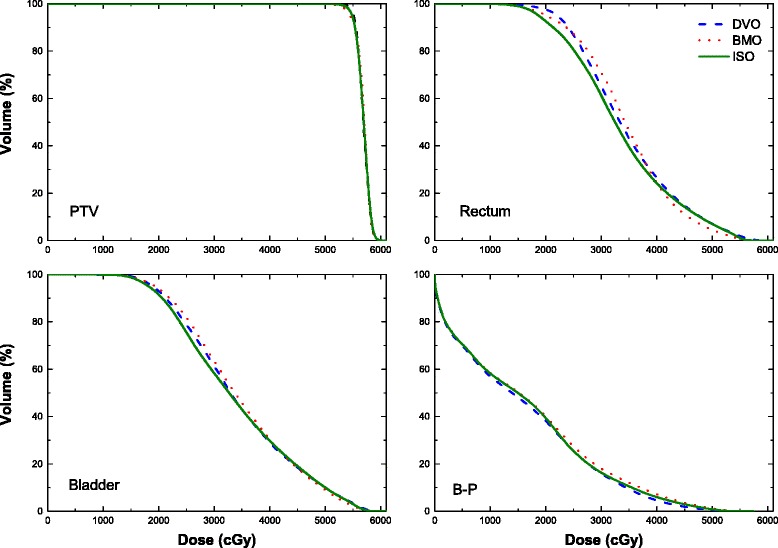
The mean DVH averaged over 20 patients of DVO, BMO and ISO plans

Figure 1 showed the cumulative DVHs, which were averaged over all patients. The PTV curves of these plans were almost overlapped. However, for the rectum, the ISO DVH curve was slightly below the DVO DVH curve and BMO DVH curve in the region from 15Gy to 42Gy. While in the region from 42Gy to 55Gy, the BMO DVH curve was lowest. As for the bladder, the same trend was observed with slightly smaller variance. For B-P, the assistance organ, the ISO DVH curve was lower than the BMO DVH curve, while higher than DVO DVH curve.

**Fig. 2:**
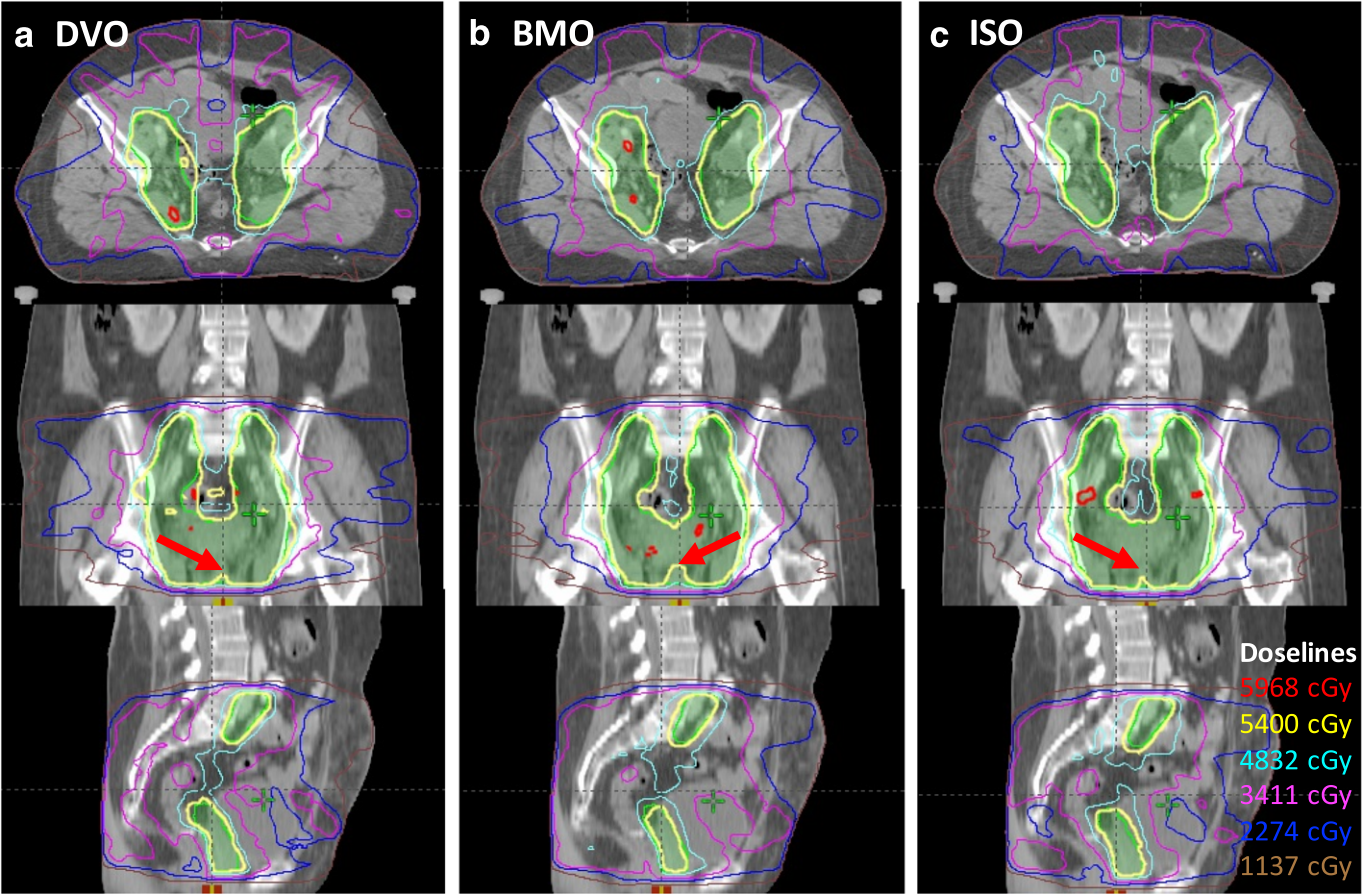
Dose distribution of one specific patient. **a** dose distribution of DVO plan; **b** dose distribution of BMO plan; **c** dose distribution of ISO plan

Dose distribution at iso-center of one specific patient was shown in Fig. 2, which represents the general condition in all cases. This figure illustrated that the dose distribution produced by BMO plans (Fig. 2.b) had more cold spots (especially in the region the red arrow indicated) in PTV than DVO plans (Fig. 2.a); whereas, in the ISO plans (Fig. 2.c), the cold spots could be reduced as the DVO plans.

## Discussion

### Comparison between DVO plans and BMO plans

Previous studies illustrated that IMRT plans optimized by biological constraints would generally more spare parallel OARs while maintaining the very similar target coverage, compared to those optimized by physical constraints [11, 12, 15, 16, 18]. In this study, compared with DVO, it was reported that using BMO would result in slightly inhomogeneous target volume and lack coverage of PTV, which could be deduced from the HI and ${V}_{95}$ in Table 4.

The TCP value, both from BMO and DVO plans, were comparable. From the equation of TCP model, it could be deduced that the same prescribed dose (including same numbers of fraction and dose per fraction) was the reason that the comparable TCP value were produced by these two optimizations. But the significant differences were observed in the physical indices, such as V_95_, D_98_, CI and HI. This may be due to the TCP model, which was used in the biological planning module, was just effectively maximizing the target-cell killing but not sensitive to the local hot spots, cold spots or the specific dose distribution. Other research studies have produced similar results [11]. On the other hand, since the brachytherapy was not considered in this study, TCP values were slightly smaller (<16) than usual. In other words, the dose received by target was insufficient. The NTCP values of OARs were also very small, which could let the biological constraints slightly effect the optimization.

Some previous studies show that adding certain physical constrains into the biological optimization process might be helpful to improve the target dose homogeneity and conformity [34]. However, Kan et. al reported that the biological planning module produces inferior target dose homogeneity with more hot spots than the DVO plans, even with physical constraints [18]. In our study, two additional physical constraints, namely, the Uniformity constraint and the Max Dose constraint, were applied for PTV in the biological planning module. This was not as effective as hypothesized. It is possible that the priority of constraints could not be modified in the biological planning module of Eclipse system. Similar limitations are discussed in previous research [18].

The NTCP of femur-heads were not referred in the results and discussion section. Because the NCTP values were 0 in all treatment plans.

On the other hand, two major structures were evaluated and compared, including bladder and rectum. Although the advantage of BMO plans was not obvious compared with DVO plans, according to the physical indices (such as V_20_, V_30_, V_40_). BMO plans could produce lower NTCP values than DVO plans (shown in Table 4). Results demonstrated that BMO could more spare rectum and bladder than conventional DVO, similar to previous research of other tumor sites [11, 12, 15, 16].

Based on the comparison of DVO and BMO plans, the min and max dose constraints were sufficient in achieving the clinical requirements in PTV coverage and homogeneity. Compared with BMO plans, these physical constraints had advantage in controlling the dose of local points. Thus, setting the physical constraints for PTV was primarily more efficient and effective. Whereas, for OARs, especially parallel OARs, the setting of physical constraints was more dependent on the planner’s experience and skills. The disadvantage of the constraints was evident when controlling the DVH trend. While biological constraints focusing on the dose received by the whole structure, would be crucial to spare the parallel OARs. Therefore, it is essential for the biological constraints to be the primary consideration for OARs.

### Different biological models

Results showed that the different NTCP models for one OAR produce the similar dose distribution. The small NTCP values of OARs (<1), mean the optimization was slightly effected by different biological constraints. Based on this point, only LKB model “ar” for rectum and LKB model “ab” for bladder were implemented in the comparison of DVO, BMO and ISO plans. However, the significant difference of relative NTCP values among different biological models (shown in Table 5) may indicate that different models significantly affect the plan quality from a biological aspect. Actually, it is a challenging work to establish a reliable and consistent biological model. Especially in cervical carcinoma, a combination of fractionated high-dose-rate brachytherapy (HDRB) and external beam radiotherapy (EBRT) should be considered [29]. And for late bladder toxicity after EBRT, there is lack of a clear dose response for whole-bladder radiotherapy [35]. And this is also the main reason that the biological optimization was mostly not implemented in clinical treatment planning. The consistency and reliability of the biological models were not specifically discussed in this work, since our study was just focus on the optimization methods comparison between biological and dose/volume approaches.

### The evaluation of ISO plans

In our study, the comparison of DVO plans and BMO plans discussed above showed that the BMO plans produce inferior coverage and homogeneity of target with more cold spots, but also more sparing of OARs. In our previous trail-and-error process, we also evaluated the plan quality that only applied biological constraints to both PTV and OARs. However, the coverage and homogeneity of PTV could not achieve the clinical requirements. Much like the BMO plans, the optimization that involved the additional physical constraints for PTV also did not produced ideal results. As for OARs; by adding physical constraints, biological constraints may be limited to reducing the probability of normal tissue complications.

Based on the above discussion, the novel strategy ISO was proposed and evaluated in this research. This differed from the process in previous studies, which proposed adding certain physical constraints into biological optimization process [4, 12, 15–18]. Via ISO strategy, our results of Figs. 1 and 2 and Table 4 show that the ISO generated comparable homogeneity and coverage of PTV as DVO plans, while more sparing OARs NTCP values as BMO plans. However, we must note that the ISO plans produce intermediate values in D_98_, V_95_, CI, HI, D_max_ and NTCP of rectum, which were directly related with optimized constraints. This demonstrated that the ISO strategy was a tradeoff between physical constraints of PTV and biological constraints of OARs. From another perspective, the least V_20_ of rectum and V_30_ of bladder generated by ISO plans indicated that these physical indices were not strongly correlated with the NTCP value. And it could be deduced D_max_, the intermediate value produced by ISO plans, was strongly correlated with NTCP value and should be highly considered in the optimization.

As for the small bowel, no significant differences were observed in all incidences, because the specific constraints for small bowel, or an accurate biology model was not implemented in the optimization process. And this is the main limitation of our work. However, BMO plans produced the highest NTCP value of small bowel, which is contrary to the rectum and bladder. And it is safety to say that the lowest NTCP values of rectum and bladder lead to this highest NTCP value of small bowel in BMO plans. This also could be induced from the similar results of B-P (Fig. 1 and Table 4).

The ISO plans quality was compromised because the priority was not assigned in the biological planning model. The physical optimization, however, was able to modify the priority and dose limits in several repeated optimization cycles [18]. If the priority could be modified for these constraints in ISO, this strategy may produce a better dose distribution and target dose homogeneity. This would be the subject of future studies.

It should be point out that OARs considered in this study were not sufficient. This is contrary to the research by Kan et al. that 7 (5 serial and 2 parallel) OARs were included in both evaluation and optimization process [18]. This is also a limitation of this study. And the ISO approach proposed in this study should be further verified in cases with more OARs and targets.

## Conclusion

In this study, BMO plans produce lower NTCP values compared to DVO plans for cervical carcinoma cases, even though this may cause slightly worse target coverage and conformity. DVO plans that apply the min dose and max dose constraints are suitable for PTV, due to its effectively in controlling the local points. On the other hand, BMO plans had an advantage in controlling the entire DVH range for OARs, especially for parallel OARs. We proposed a strategy in this study: set the physical constraints as the PTV primary option and the biological constraints as OAR primary option. By means of this strategy, the indices of PTV in ISO plans improved greater than BMO plans, and the indices of OARs were better than that of DVO plans. The fact that different models significantly affect the plan quality indicates that establishing an accurate biological model of OARs is very necessary and urgent for biological optimization.

## Abbreviations

AAA: Anisotropic analytical algorithm
CT: Computed tomography
CTV: Clinical target volume
gEUD: Generalized equivalent uniform dose
HI: Homogeneity index
IMRT: Intensity-modulated radiation therapy
NTCP: Normal tissue complication probability
OAR: Organ at risk
PTV: Planning target volume
TCP: Tumor control probability
